# Folate^☆^

**DOI:** 10.1016/j.advnut.2025.100519

**Published:** 2025-09-17

**Authors:** Zoe Lofft, Timothy J Green, Angela M Devlin

**Affiliations:** 1 Department of Pathology and Laboratory Medicine, The University of British Columbia, BC Children’s Hospital Research Institute, Vancouver, Canada; 2 College of Nursing and Health Sciences, Flinders University, Adelaide, Australia; 3 Department of Pediatrics, The University of British Columbia, BC Children’s Hospital Research Institute, Vancouver, Canada

Folates are a group of chemically related compounds that share a common structure comprising 3 moieties: a pteridine ring (in an oxidized or reduced form), para-aminobenzoic acid bridge, and mono- or polyglutamate tail [1]. Broadly, folates can be classified as naturally occurring or synthetic. Food folates are in the reduced form, predominantly tetrahydrofolate (THF), and are polyglutamated, whereas most synthetic folates, such as folic acid, are highly stable, oxidized, and monoglutamated [2]. Folates are absorbed by pH-dependent active transport across the enterocytes of the proximal small intestine and enter circulation by the portal vein [3]. For natural folates, the glutamate residues must first be hydrolyzed by the brush border membrane folate hydrolase before transport into the enterocyte. Folic acid is directly absorbed by enterocytes but it is reduced twice by dihydrofolate reductase to be converted into its active form, THF [1]. This reaction can become saturated in the enterocyte at intakes exceeding ∼200 μg, leading to a rise in unmetabolized folic acid in circulation [4]. Circulating unmetabolized folic acid concentrations are thought to be transient and dependent on the time after folic acid ingestion. However, the biological effects of unmetabolized folic acid remain unclear and are an area of investigation.

THF is converted further into methyl- or formylfolate intermediates that play a critical role in 1-carbon metabolism by carrying, transferring, and accepting 1-carbon units, leading to the generation of amino acids (serine, glycine, and methionine) for protein synthesis and nucleotides (purines and thymidine) for DNA/RNA synthesis. Folate-dependent 1-carbon metabolism is compartmentalized and takes place in the cytosol, mitochondria, and nucleus, allowing for segregation of metabolic functions [1]. The predominant folate form in circulation is 5-methyltetrahydrofolate (5MTHF). This form is required for the remethylation of homocysteine to methionine in a vitamin B12-dependent reaction catalyzed by methionine synthase. Methionine serves as the substrate for S-adenosylmethionine synthesis, which plays a key role as a methyl donor for several compounds, including nucleic acids, proteins, and phospholipids [2]. There is some evidence that folate is produced by select bacterial species and yeast in the gut microbiota, but it is concomitantly consumed by folate auxotrophs, and it is unknown if it contributes substantially to folate status [5].

## Deficiencies

Folate status is defined by circulating folate concentrations. Serum total folate is reflective of recent dietary intakes, and erythrocyte total folate concentrations represent the past ∼120 d, when folate was incorporated during erythropoiesis. The gold standard quantification method for erythrocyte folate is a microbiological assay using *Lactobacillus casei* [6]. According to the WHO population guidelines, folate deficiency is defined as serum folate <6.8 nmol/L and/or erythrocyte folate <226.5 nmol/L [6]. Classical folate deficiency presents as macrocytic anemia (large, abnormally nucleated erythrocytes). Elevated homocysteine is a marker of folate deficiency because 5MTHF is required by methionine synthase for the remethylation of homocysteine to methionine. However, the reaction also requires vitamin B12, and homocysteine concentrations become elevated under conditions of low folate and/or low vitamin B12 status. Therefore, elevated homocysteine is only a marker of folate status under conditions of adequate vitamin B12 status. Elevated homocysteine is also associated with distinct, unrelated conditions, including impaired kidney function, and concentrations change during physiological adaptations, such as those that occur during pregnancy [2]. In vitamin B12 deficiency, folate becomes functionally trapped as 5MTHF, preventing its conversion back to THF. This occurs because the remethylation of homocysteine to methionine—catalyzed by methionine synthase—requires both 5MTHF and vitamin B12. In the absence of vitamin B12, this reaction is impaired, and 5MTHF accumulates. As a result, THF is not regenerated, and its functions, such as those in nucleotide synthesis and mitochondrial metabolism, are compromised [1].

Dietary folate requirements change throughout the lifespan, with increased requirements during rapid growth and development to support essential biosynthetic processes and cell division. In the developing fetus, maternal folate deficiency is associated with neural tube defects (spina bifida, anencephaly, or encephalocele) caused by the inability of the neural tube to fully close, which occurs in the first month of pregnancy, and/or other congenital anomalies. **A** daily folic acid supplement, typically ∼400 μg/d, is still recommended for women of childbearing age to reduce the risk of neural tube defects [7].

## Diet Recommendations

Requirements for folate are expressed as dietary folate equivalents (DFEs) to adjust for differences in bioavailability between natural and synthetic folate forms. Folic acid is ≤100% bioavailable when consumed without food, whereas folates in food are ∼50% bioavailable relative to folic acid [3]. Bioavailability may be reduced when folic acid is consumed with food or when natural folate is not released from the food matrix [5].

DFEs are calculated using the following formula: DFE (μg) = μg of food folate + (1.7 × μg of folic acid). The recommended dietary allowance for folate, expressed in DFEs, changes over the lifespan: 150 μg for ages 1–3 y, 200 μg for ages 4–8 y, 300 μg for ages 9–13 y, 400 μg for ages 14–18 y, and 400 μg for adults aged >19 y. Dietary folate recommendations are highest during pregnancy (600 μg) to support rapid cell division as the fetus develops and during lactation (500 μg) to account for losses in human milk [3].

## Food Sources

Folate is found naturally in dark leafy green vegetables, legumes, whole grains, and citrus fruits [2]. Losses of natural folates can occur during food preparation. Folate is vulnerable to leaching into cooking water and oxidative degradation when heating food [5]. In 1998, in an effort to prevent neural tube defects, the United States Food and Drug Administration mandated the fortification of enriched breads, cereals, flours, pasta, and other grain products with folic acid (140 μg/100 g) [2]. Since then, over 70 countries have implemented folic acid fortification programs, which have successfully reduced neural tube defects [8]. At a population level, folate deficiency is rare in countries with fortification policies [9].

## Clinical Uses

Higher doses (1–5 mg) of folic acid have been recommended to women at increased risk of having a pregnancy affected by neural tube defect or other folic acid-sensitive congenital anomalies. Risk factors may include a previous neural tube defect-affected pregnancy, pregestational diabetes, obesity, or taking a medication that may interfere with folic acid absorption and/or metabolism (methotrexate, antiepileptic medications, and sulfasalazine) [7]. Newer guidelines have proposed taking an individualized approach by evaluating folate status in reference to levels known to be protective against folic acid-sensitive neural tube defects (fasting serum folate 28–30 nmol/L; red blood cell folate >907 nmol/L) [7]. The personalization of recommendations pertains to both the dose (0.4 mg, standard; 1 mg, moderate; 4–5 mg, high dose), which is modified relative to neural tube defect risk, and duration. In some cases, a moderate- or high-dose supplement may only be recommended during the preconception period to the first 12 weeks of gestation before switching back to the standard (0.4 mg) dose throughout the remainder of pregnancy and lactation [7].

## Toxicity

A tolerable upper intake level (UL) of 1 mg/d for adults has been established for folic acid (supplements and fortified foods) but not for other forms of folate. No adverse events have been associated with intake of folate found naturally in foods. The determinant for setting the UL was over the concern of masking vitamin B12 deficiency, given that it also presents as macrocytic anemia [3]. This is significant because irreversible neurological complications can result from prolonged vitamin B12 deficiency.

## Recent Research

Current research is focused on understanding the long-term effect of folic acid fortification, after the successful impact of the strategy to reduce neural tube defects [8], and the biological effects of both high folic acid intakes and unmetabolized folic acid [10]. Unmetabolized folic acid in serum has been found in children and adults in NHANES data post fortification [11]. Further, unmetabolized folic acid has been detected in breastmilk and umbilical cord blood, suggesting that fetuses and breastfed infants are exposed to it [12,13]. The presence of unmetabolized folic acid in breast milk suggests that it is taken up but not metabolized by mammary tissue. Additional research is required to fully understand the biological effects of higher than recommended folic acid intakes and how folic acid is metabolized by different tissues.

There is also ongoing interest in the relationship between high folate intake and metabolic health. A meta-analysis of 29 randomized controlled trials (*n* = 27 folic acid; *n* = 2 5MTHF; 0.4–15 mg/d) in adult males and females found that folate supplementation decreased fasting insulin and insulin resistance, estimated by HOMA-IR, but had no effect on fasting glucose, glycated hemoglobin (HbA1c), or risk for type 2 diabetes [14]. The studies included in the analyses varied greatly in age, sex, and underlying metabolic status of the participants before the intervention. A more recent double-blind randomized controlled trial in males and females (aged 45–75 y; *n* = 100) with type 2 diabetes receiving metformin found that folic acid (5 mg/d, 12 wk) lowered fasting blood glucose, HbA1c, and HOMA-IR [15]. Both studies revealed accompanying decreases in homocysteine and highlighted lowered homocysteine as a mediator of improvements in glucose metabolism. The inverse relationship between homocysteine and insulin resistance has previously been postulated [16]. Classically, homocysteine is known to induce oxidative stress [17], to which insulin-producing β cells in the pancreas are highly sensitive [18]. Mechanistically, recent intricate preclinical research has shown that homocysteine can inhibit proinsulin receptor cleavage, leading to insulin resistance [19], and inhibit β-cell insulin secretory function [20].

Despite some evidence of a positive effect of folate supplementation on metabolic health in adults, discordant findings have been reported on the relationship between high folate status/folic acid intake and the development of gestational diabetes mellitus. A prospective cohort study in China (*n* = 4353) found that folic acid supplementation ≥0.8 mg/d from prepregnancy through midpregnancy was associated with an increased odds ratio (OR) of gestational diabetes, compared with those who did not supplement [OR: 2.36; 95% confidence interval (CI): 1.51, 3.69] [21]. Conversely, findings from the Nurses’ Health Study II (*n* = 14,553) showed that higher supplemental folate intake was inversely associated with the relative risk (RR) of gestational diabetes, compared with those who did not supplement (RR for ≥0.6 mg/d: 0.70; 95% CI: 0.52, 0.94) [22]. Interestingly, naturally occurring food folate was not associated with gestational diabetes risk. More recently, a U-shaped relationship between folic acid supplementation and gestational diabetes diagnosed by oral glucose tolerance test was observed in a case-control study in China (*n* = 1300). Compared with those who supplemented with 0.4–0.799 mg/d folic acid before and during pregnancy, those who did not supplement and those who supplemented with ≥0.8 mg/d had increased risk of developing gestational diabetes (no supplement, OR: 3.28; 95% CI: 1.08, 9.96; ≥0.8 mg/d, OR: 2.88; 95% CI: 1.94, 4.28) [23].

Although the aforementioned studies relied on self-reported dietary intake, biochemical folate measurements, such as serum folate, can surmount the self-reporting limitations and provide a current depiction of folate stores. A recently published longitudinal assessment in pregnant women in western India, with and without gestational diabetes (*n* = 100/group), found higher circulating folate levels and folate supplementation practices in those with gestational diabetes, despite comparable intakes of folate-rich foods [24]. Interestingly, gestational timing was a salient factor, as higher folate status was only associated with increased gestational diabetes risk beyond 18 weeks of gestation [24]. When synthesizing the evidence, heterogeneity in study design pertaining to the measurement of folate intake and/or status, duration of supplementation, consideration of gestational timing, and criteria for gestational diabetes diagnosis often precludes effective comparisons between findings.

Further research is required to delineate the effect of high folate status and unmetabolized folic acid on glucose homeostasis and the pathophysiology of type 2 diabetes and gestational diabetes. Although supplemental folate remains necessary during pregnancy, 5MTHF has been shown to be equally effective as folic acid in maintaining folate status during pregnancy while circumventing elevations in unmetabolized folic acid [25]. Continued study into the molecular and metabolic effects of high folate status and consuming folate in different forms will further enhance our understanding of optimal intake and supplementation guidelines throughout the life course.

For more information, refer Refs. [3,8]

## Author contributions

The authors’ responsibilities were as follows – all authors: read and approved the final manuscript.

## Funding

AMD was supported by funding from the Canadian Institutes of Health Research.

## Conflict of interest

AMD reports financial support was provided by BC Children’s Hospital Research Institute. If there are other authors, they declare that they have no known competing financial interests or personal relationships that could have appeared to influence the work reported in this paper.
